# Supplementary material for: Pain and the emotional brain: pain-related cortical processes are better reflected by affective evaluation than by cognitive evaluation
Source: Sci Rep. 2023 May 22;13:8273. doi: 10.1038/s41598-023-35294-2 (PMC10202916; doi:10.1038/s41598-023-35294-2)
Supplement: Supplementary file 2 — Supplementary Information. [file 41598_2023_35294_MOESM2_ESM.pdf]

Improved statistical modelling using a gradient vector

Regarding the BOLD data, we have adopted a more suitable approach, which we have now included in the supplement since it has often gone misunderstood by reviewers. We constructed a vector, UI_gradient, across all trials (modulated and unmodulated), which represents the difference between pain intensity and pain unpleasantness for each individual trial. This gradient is more effective in distinguishing the encoding of pain intensity and pain unpleasantness, as it assigns greater weight to larger differences between the two pain descriptors. A value of zero is used to indicate no difference. Conversely, the conventional contrast ([-1 1], as shown in Supplementary Table 2 in orange), which was used in the main article, fails to distinguish between trials with substantial and no differences in the rating of both pain descriptors.
The gradient vector was created for each trial as follows:

(1) UI_gradient = [(U-I)/2; (I-U)/2]

In addition, we have taken steps to account for any unexplained variance that could potentially affect the distinction between intensity encoding and unpleasantness encoding. The inclusion of the variable "mean_rating" will help control for any impact of the magnitude of pain ratings. Specifically, the level of experienced pain (M=(U+I)/2) may influence the difference between intensity encoding and unpleasantness encoding. Moreover, we have included the variable "condition_mean," which provides information on the average rating for each condition (reappraisal, counting, imagination, pain only), descriptor, and subject. As a result, we have adapted the formula as follows:

(2) rating ~ fmri + mean_rating + condition_mean + *UI_gradient:fmri + (1|subject)*

Naturally, as intensity and unpleasantness are highly correlated the variable “mean_rating” has a highly significant effect (t=16.95, p<0.001 for a right insular voxel). The “condition_mean” has no effect (all p>0.05). The results exhibit a better model fit compared to the model in the main article as represented by higher t-values. The statistical threshold is adjusted to account for multiple testings (p<0.05; PALM).

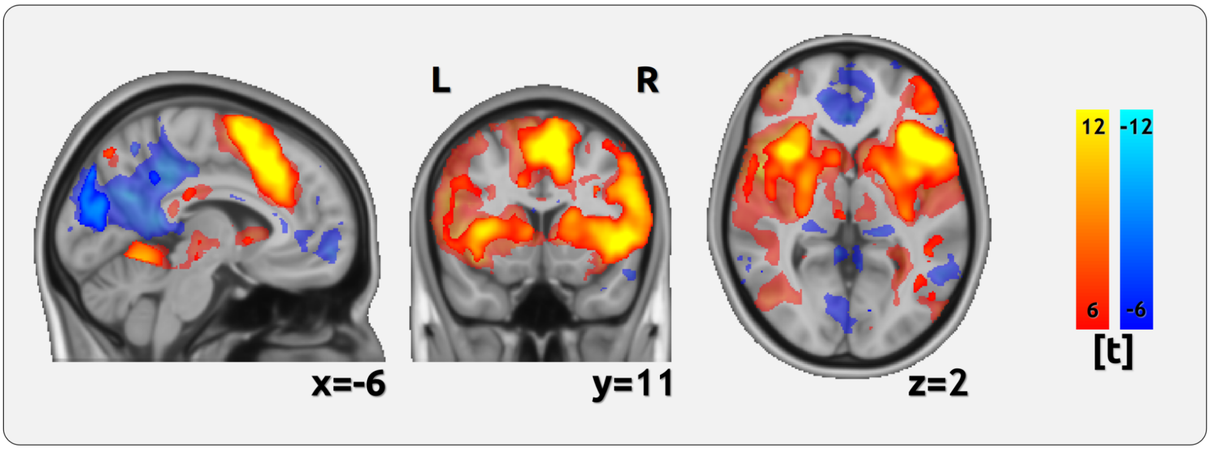

Supplementary Figure 1 | Differences between pain unpleasantness encoding and pain intensity encoding. Several cortical regions reflect the gradual differences between pain intensity

and pain unpleasantness. Warm colours indicate regions that show a more positive relationship for pain unpleasantness; cold colours indicate brain regions that show a stronger negative relationship for pain unpleasantness. In order to better separate the different regions, we increased the statistical threshold beyond the significance level (t>4.9) for the positive effects. The pale colours indicate the contrast between pain trials and baseline (t>2).

Results

max t-value	x	y	z	region
13.5	-45	14	-2	Inferior Frontal Gyrus
13.5	-9	14	47	Paracingulate Gyrus
-9.13	2	-89	24	Precuneous Cortex
13.4	35	15	5	Insular Cortex
11.6	26	-55	47	Superior Parietal Lobule
11.4	-43	-40	48	Superior Parietal Lobule
10.1	37	-24	54	Precentral Gyrus
10.3	48	5	35	Precentral Gyrus
11	-10	-53	-12	Lingual Gyrus
9.5	43	29	31	Middle Frontal Gyrus
-9.02	26	33	44	Superior Frontal Gyrus
10.2	29	51	15	Frontal Pole
9.73	-33	-18	-2	Insular Cortex
9.98	-60	-19	26	Postcentral Gyrus
9.23	-10	-72	46	Precuneous Cortex
-5.77	0	60	-8	Frontal Pole
-6.34	59	-55	5	Middle Temporal Gyrus
-5.96	38	-72	27	Lateral Occipital Cortex
-7.26	26	-4	-18	Amygdala

Supplementary Table 1. Differences in the encoding of pain intensity and pain unpleasantness. Positive effects indicate a stronger positive relationship for unpleasantness; negative relationships indicate a stronger negative relationship for unpleasantness (all p<0.05, PALM corrected).

Data structure of one exemplary voxel.

subject	trial	rating	fMRI	rating_type	rating_code	gradient
1	1	25	30.91	intens	-1	-2.5
1	1	30	30.91	unpleas	1	2.5
2	1	25	124.92	intens	-1	5
2	1	15	124.92	unpleas	1	-5
3	1	25	61.30	intens	-1	7.5
3	1	10	61.30	unpleas	1	-7.5
4	1	15	23.53	intens	-1	2.5
4	1	10	23.53	unpleas	1	-2.5
5	1	35	75.44	intens	-1	5
5	1	25	75.44	unpleas	1	-5
6	1	10	50.15	intens	-1	2.5
6	1	5	50.15	unpleas	1	-2.5
7	1	10	56.58	intens	-1	2.5
7	1	5	56.58	unpleas	1	-2.5
8	1	30	22.63	intens	-1	-7.5
8	1	45	22.63	unpleas	1	7.5
9	1	50	53.17	intens	-1	0
9	1	50	53.17	unpleas	1	0
...						
11	44	35	57.02	intens	-1	2.5
11	44	30	57.02	unpleas	1	-2.5
12	44	10	-53.56	intens	-1	0
12	44	10	-53.56	unpleas	1	0
13	44	35	120.45	intens	-1	2.5
13	44	30	120.45	unpleas	1	-2.5
14	44	45	40.20	intens	-1	0
14	44	45	40.20	unpleas	1	0
15	44	40	143.39	intens	-1	0
15	44	40	143.39	unpleas	1	0
16	44	30	41.96	intens	-1	2.5
16	44	25	41.96	unpleas	1	-2.5
17	44	50	85.72	intens	-1	-5
17	44	60	85.72	unpleas	1	5
18	44	55	181.06	intens	-1	-5
18	44	65	181.06	unpleas	1	5
19	44	35	-10.43	intens	-1	-2.5
19	44	40	-10.43	unpleas	1	2.5
20	44	20	3.32	intens	-1	0
20	44	20	3.32	unpleas	1	0

Supplementary Table 2 | Please note the “mirrored” values of the gradient. It shows that the gradient approach is more suitable for the model as higher gradient values correspond with larger differences between the ratings of intensity and unpleasantness. This gradient score “weighs” the differences between both pain measures (compare trial 1 of subjects 1 and 2). The dichotomous model does not make any difference in the contrast of both measures. The dichotomous model compares all trials equally, even if there is no difference in rating for intensity and unpleasantness.
